# Integrative nursing interventions: knowledge, attitudes and practice in home nursing services in Germany—a quantitative and qualitative online survey

**DOI:** 10.3389/fmed.2024.1438035

**Published:** 2024-10-03

**Authors:** Regina Stolz, Carina Klocke, Cornelia Mahler, Jan Valentini, Stefanie Joos

**Affiliations:** ^1^Institute of General Practice and Interprofessional Care, University Hospital and Faculty of Medicine, Tübingen, Germany; ^2^Department of Nursing Science, Institute for Health Sciences, University Hospital and Faculty of Medicine, Tübingen, Germany

**Keywords:** integrative nursing, self-care, home nursing service, Naturheilkunde, non-pharmacological interventions, primary health care

## Abstract

**Introduction:**

Integrative nursing interventions (INI) play a significant role in healthcare, particularly in the prevention and treatment of chronic diseases. Integrating evidence-based INI into healthcare aligns with global initiatives such as the WHO’s Decade of Healthy Aging 2020–2030. Many INI are low-threshold practices, empowering patients to independently manage health. However, the extent to which INI are used by home-care nursing-services (HNS) remains largely unknown. This study aims to explore the field of INI in German HNS regarding nurses’ use of INI as well as attitudes, subjective knowledge, and information needs on the subject.

**Methods:**

A cross-sectional anonymous online survey with 29 Likert scale items and two open-ended questions was conducted between April 2023 and July 2023. The survey targeted nurse managers of HNS in Baden-Württemberg, Germany. Descriptive analysis was performed for quantitative data, while content analysis according to Kuckartz was applied to analyze open-ended text responses.

**Results:**

In total, *n* = 68 out of *n* = 1,331 HNS took part in the survey yielding a response rate of 5.1%. Their overall attitude toward INI was clearly positive (10-point Likert scale M ± SD: 8.37 ± 2.22). The average self-assessed knowledge level about INI was moderate (M ± SD: 5.39 ± 2.76). Almost half of the participants (45.6%) declared to incorporate INI in patient care. Most participants (84.2%) lacked employees with additional qualifications in INI. The INI used most were medicinal herbal teas (61%), compresses (57%), and aromatherapy (48%). Acupressure showed the greatest disparity between actual use in participating HNS (4.3%) and interest in further education (61%). The most common symptoms for which INI are used are pain, respiratory problems, anxiety, and palliative care. The main challenges reported for the use of INI in HNS are financial aspects, qualification and limited resources (staff and time).

**Discussion:**

This exploratory study provides the first insights into nurses’ attitudes, self-assessed knowledge, and utilization of INI in German HNS. Overall response rate was low (5.1%), therefore, the results should be interpreted with caution. Urgent action is needed to address financial aspects and further education on INI, to promote integration of INI in HNS to the best possible extent.

## 1 Introduction

Integrative nursing interventions (INI) play a significant role in global healthcare, particularly for the prevention and treatment of chronic disease (1, 2). This recognition is endorsed by the World Health Organization (WHO) (3, 4). Integrating evidence-based INI into healthcare aligns with global initiatives such as the WHO’s Decade of Healthy Aging 2020–2030 (5, 6).

Nurses are approached by their patients for INI and increasingly apply them as complementary interventions during standard patient care (7). One reason for this is that they are considered low-threshold, safe interventions that can be learned by patients and their relatives (8–11). Kligler et al. discovered that employing”effective educational strategies that encourage patient engagement in symptom self-management and strategies that improve whole-person wellbeing” are considered profession-specific nursing competencies in the field of Integrative Health (12).

To date, there is no clearly defined terminology for interventions considered complementary to biomedical practice and nursing (1). Terms like “Integrative Medicine” (IM), “Integrative Nursing” (IN) or “Complementary and Integrative Health” (CIH) are used to refer to evidence-based approaches to implementing these interventions in health care (1, 13–15). In German-speaking countries, the term “Naturheilkunde” is commonly used in the context of CIH, especially for nursing interventions of German Traditional European Medicine (TEM) (16, 17). For consistency in terminology, the term “complementary and integrative health” (CIH) will be used throughout this study, even when referring to studies that use the term “complementary and alternative medicine” (CAM) or “complementary and integrative medicine.” The term “integrative nursing interventions” (INI) will be used to describe CIH interventions that are applicable to nursing practice.

INI encompasses therapies such as wraps, compresses, aromatherapy, and acupressure, therefore, taking a holistic approach to healthcare by addressing physical, psychological, social, and spiritual dimensions (18–20). Many INI facilitate self-care practices, empowering patients to manage their health independently, either with guidance from healthcare professionals or autonomously (21). As a result INI are an important resource for self-care, with patients’ confidence in their ability to care for themselves being a strong predictor of physical and emotional quality of life (22). Numerous studies have shown the effective promotion of (older) people’s self-care skills by professional caregivers (7, 23–28).

Self-care skills can be helpful for people in need of long-term care. In Germany, more than 80 percent of people in need of long-term care are cared for at home, with more than half (51.3%) being cared for by family caregivers and with around a third (36%) being supported by home nursing services (HNS). HNS play an increasingly important role in supporting family caregivers (29). Furthermore, INI such as acupuncture/acupressure and phytotherapy have accumulated evidence from randomized controlled trials and meta-analyses that supports their efficacy and relevance in clinical practice (30, 31). The German S3 guideline for complementary methods in oncology underscores the growing recognition of these interventions (32).

For external applications, which are the main INI, there is a growing body of evidence for their efficacy. For example, studies have shown the effectiveness of topical herbal medicines for treating psoriasis (33), hot abdominal compresses for increasing liver blood flow (34), yarrow liver compresses for reducing fatigue in cancer patients (35), cabbage leaf wraps for osteoarthritis of the knee (36), and footbaths with mustard, ginger, or warm water alone for improving heat distribution (37). However, there is a lack of studies of external applications, therefore expert consensus provides the best available evidence in these cases (16).

The strong patient preference for INI also plays an important role in evidence-based healthcare (38, 39). Studies show a high level of acceptance of INI and CIH among patients (8, 15, 21, 40–42). In Europe 25.9% of the general population reported using CIH, with two to four times higher usage among those with health problems compared to those in good health (43). In Switzerland, around a third of the total population report using CIH, while in Germany the figure ranges from 40 to 62% (44–46). Patients use INI and CIH for various reasons, among them expecting benefits in treating their diseases and alleviating the side effects of conventional therapy (9, 47, 48).

In Germany, the growing demand for INI is contrasted by the lack of formalized training for these skills. Although there are many INI education and training options available, they vary widely in eligibility requirements, length, content, evidence base, certification, and cost. An evidence-based formalized education on CIH and INI is also important for patient safety, as asserted by the WHO in 2013, which emphasized that the knowledge and qualifications of practitioners directly impact patient safety (4).

Nurses are often very open to CIH (49), particularly in oncology, where they see CIH as an opportunity to provide more individualized care to patients. Nurses report that CIH enables them to expand their nursing practice, feel more satisfied with their work and have an effective tool for empowerment (50). Data from a survey of health care professionals at the four university hospitals in Baden-Württemberg show a high level of acceptance of CIH and INI among nurses, even higher than that of medical staff (15). Similarly, a survey of members of the German Society for Palliative Medicine found a high acceptance of CIH and INI (92%) and noted differences between healthcare professions (51). CIH is already widely used in general practice in Germany where approximately 60% of family doctors use CIH procedures such as phytotherapy, acupuncture, and neural therapy (52).

For the German-speaking primary care setting, no INI data from nurses working in HNS could be found in the literature. Therefore, this study aims to gain initial insights into the perspectives of nurses in HNS in Germany on the use of INI, their attitudes, knowledge, and the need for information on the topic of INI.

## 2 Methods

### 2.1 Research questions

To gain insights into the field of INI in German HNS regarding nurses’ use of INI as well as their attitudes, current knowledge and perceived need for continuing education on the subject the following research questions were specifically addressed:

1. Status quo in daily care: To what extent are INI offered in HNS in Baden-Württemberg?
2. Attitudes and knowledge of providers: What are the attitudes and knowledge of nurses working in HNS toward INI?
3. Spectrum: Which INI are specifically used by HNS nurses to prevent or treat a patient’s symptoms and health complaints?
4. Interest in training: What skills and information do HNS nurses need regarding INI?
5. Enablers and barriers: What are the enablers and barriers to the use of INI in HNS?

### 2.2 Study design

We conducted an explorative cross-sectional online survey between April and July 2023 using Unipark software (Questback GmbH). The survey was anonymous and addressed the nurse managers of all 1,331 HNS in the federal state of Baden-Württemberg, Germany. According to the Ethics Committee of the University Hospital and Faculty of Medicine of Tübingen, and in accordance with the German Federal Law § 3 Abs. 6 BDSG/LDSG BW, no formal ethics approval is required for the collection of anonymous data. Therefore, the Ethics Committee of the University Hospital and Faculty of Medicine Tübingen did not provide a consultation. The study was conducted in accordance with local legislation and institutional requirements. The participants gave their written informed consent to participate in this study. To ensure anonymity, no IP addresses were recorded and no cookies were used. The survey targeted nurses with leadership roles in the HNS, as they can provide comprehensive information on the use of INI and the challenges involved, including the economic perspective.

To date, no suitable validated questionnaire has been found in the literature. Therefore, we developed a questionnaire based on our research questions. As a basis, we used a questionnaire recently distributed to healthcare professionals at university hospitals in Germany (15), and a questionnaire used in a nationwide survey of general practitioners in Germany (52).

We then adapted it to the context of HNS. The questions adopted from the previous questionnaires pertain to general attitudes toward INI, attitudes toward INI in HNS, knowledge of INI, and training needs (research questions 2 and 4). The questionnaire underwent a two-stage pretest. Initially, it was pretested by a group of eight health-care professionals in a methods workshop. Subsequently, it was cognitively pretested by five nurses of the target group with the think aloud method (53) for content completeness, clarity, and duration of the survey. The nurses are part of the author’s research network. All hold managerial positions and have work experience in HNS, with three having additional qualifications in INI. After pretesting, the questionnaire was adapted accordingly.

The final version of the questionnaire contained 29 Likert scale questions (for research questions 1, 2, and 4) and two open-ended questions (for research questions 3 and 5).

The 29 Likert scale questions are divided into six sections covering attitudes, practice of INI in HNS, knowledge and information needs, socio-demographic data, characteristics of HNS and challenges. The scales include 5- and 10-point endpoint Likert scales, e.g., on general attitudes toward INI (10 = “very favorable”) or motivation to use INI in HNS (5 = “I fully agree”), as well as multiple-choice and single-choice questions.

In addition, a list of nine INI was used to assess the methods used in HNS, the additional qualification of participants and nurses, and the need for continuing education. These nine items were derived from previous studies (7, 10, 54). Due to the exploratory nature of the study, two additional questions were open-ended (reason for using INI in HNS, challenges associated with using INI in HNS) and six questions allowed for free-text responses in the “other” field (e.g., reasons for not using, reimbursement options, INI used).

### 2.3 Recruitment of participants

The survey was conducted as a full survey. However, there is no publicly available directory of all HNS in Germany. Since all services are organized through providers, they were asked to distribute the survey link to their members starting in April 2023. Of 11 providers contacted, three declined to distribute the link, citing restrictions on external links due to data security issues. Therefore, a list was manually compiled based on the AOK-Pflegenavigator (as of May 7th, 2023), an online database of a large health insurance company that lists all HNS. The survey link was then distributed to all 1,331 listed HNS in the state of Baden-Württemberg, Germany, in June 2023. For previously contacted HNS, this email served as the initial reminder, followed by reminders sent to all 29 email addresses that send out-of-office notifications. The survey was completed after 12 weeks in July 2023, 2 weeks after the last recorded end of leave date according to participants’ out-of-office notifications.

As an incentive to participate in the survey, a free online training course on evidence-based INI in HNS (90 min) was offered to all staff at the participating HNS.

### 2.4 Analyzed data

All questionnaires that received an answer “yes” or “no” to the only mandatory question “Do you use INI in your home nursing service?” were included in the analysis with variable n due to missing data. The number of responses to each item was variable due to missing data. Cases were excluded only if the duration was implausible (e.g., survey time < 1 min, *n* = 6). Several plausibility checks were performed, e.g., to ensure that no service participated twice [e.g., plausibility of number of staff (number of variable (v)101), organization (v164), location (v100)] or to control for a non-response bias.

Two variables were recoded for analysis. “Year of exam” (v_78) was recoded to “Years since exam” (v_78_new), HNS provider (v_164 v_165) was recoded to provider status (private, non-profit, public; v_164 v165_new).

### 2.5 Analysis

IBM SPSS Statistics 28 was used for descriptive statistical analysis. Results are presented as absolute frequencies and percentages or as mean ± standard deviation (SD).

All written responses to the two open-ended questions were analyzed using content analysis following Kuckartz’s approach, to generate major categories and their corresponding subcategories (55). Responses to the six questions that allowed free-text responses in the “other” field were analyzed descriptively.

Two investigators developed a first coding system of thematic categories. Through consensus, initial codes were refined to create a coherent system of categories. The emergent themes from the content analysis were then discussed with the other data of the survey.

Participants’ quotes were translated from German into English and are presented along with their pseudonyms to highlight the findings in this article.

Data reporting in this article follows the standard of the consensus-based Checklist for Reporting of Survey Studies (CROSS) (56).

## 3 Results

### 3.1 Response rate

The survey link was sent to *n* = 1,331 HNS. The overall response rate was 5.1% (*n* = 68).

### 3.2 Sociodemographic characteristics of participants

Sociodemographic data are summarized in Table 1. Not all 68 participants answered all the questions. The gender question was answered by 57 participants and of which four fifths (80.7%, *n* = 46) identified as female. The majority (91.2%, *n* = 52) had a working capacity of 75 to 100% (part time employment measure where 100% would be considered full time employment).

**Table 1:** Demographic categories.

Demographic categories	N	%
Gender (*n* = 57 respondents)		
Female	46	80.7
Male	11	19.3
Nursing qualification (*n* = 59)^1^		
Registered nurse (3 year qualification with state exam)	53	89.8
Postgraduate bachelor degree in nursing science	11	18.6
Other postgraduate academic qualification^2^	5	8.5
Leadership position (*n* = 59)		
Head of nursing service	38	64.4
Deputy head of nursing service	12	20.3
Management	14	23.7
Other^3^	8	13.6
No leadership position	1	1.7
Involved in direct patient care (*n* = 57)		
Yes	55	96.5
No	2	3.5
Personal use of INI (*n* = 66)		
Yes	57	86.4
No	9	13.6
Percentage of work^4^ (*n* = 57)		
< 50%	2	3.5
50–75%	3	5.3
75–100%	52	91.2
	Mean	SD
Age, in years (*n* = 56)	49.46	11.91
Years since exam *n* = 54	25.69	12.73

Demographic categories with frequency (N) and valid percentage (%).

1 Nursing education in Germany is predominantly vocational, with few bachelor’s degree programs. Some nurses also pursue a bachelor’s or master’s degree in nursing science.

2 e.g., public health, palliative care MAS.

3 Other (e.g. quality management, continuing education and training, clinical supervisor).

4 Where 100% corresponds to full-time employment.

The mean age of the participants was 49.5 years (SD = 11.9), and they had an average of 25.7 years (SD = 12.7) of experience since graduating from nursing school. Almost all respondents (96.5, *n* = 57) were involved in direct patient care. The majority of respondents/nurses (86.4%, *n* = 57) use INI personally.

### 3.3 INI in HNS

#### 3.3.1 Use of INI and patient demand for INI

The question “Do you use INI in HNS” was the only mandatory question, meaning that all *n* = 68 participants provided an answer. Slightly more than half (54%, *n* = 37) of the HNS do not use INI, while 46% (*n* = 31) do. Patients’ demand for INI (question: “I myself am explicitly asked about INI by patients”) was answered by *n* = 57 participants, whereby 63.2% (*n* = 36) with “never or rarely (about once every 6 months)” and 15.8% (*n* = 9) with “very often (several times a week)” or “frequently (about once per week).”

#### 3.3.2 General attitude toward INI

The general attitude of the responding nurse managers (*n* = 65) toward INI (question: “What is your general attitude toward INI”) was clearly positive (*M ± SD*: 8.37 ± 2.219; Likert scale: 1 = “very unfavorable,” 10 = “very favorable”).

#### 3.3.3 Characteristics of HNS

The majority of HNS (84.2%, *n* = 48) did not have staff with additional qualification in INI.

All HNS in Germany have a provider that has one of the following statuses: private/for-profit, non-profit (e.g., charitable and religious associations), or public (municipalities, municipal associations). When asked about the type of provider, nearly two-thirds of participants (27/44 = 61.4%) indicated a private provider, one-third non-profit provider (15/44 = 34.1%), and (2/44 = 4.5%) a public provider. See Table 2 for more information.

**Table 2:** Characteristics of HNS.

	N	%
Provider (*n* = 44)^1^		
Private/ for-profit	27	61.4
Non-profit	15	34.1
public	2	4.5
Use of INI in the HNS (*n* = 68)		
Yes	31	45.6
No	37	54.4
Additional qualification INI (leadership) (*n* = 57)		
Yes	8	14.0
No	49	86.0
Employees with additional INI qualification (*n* = 57)		
Yes	9	15.8
No	48	84.2
Location of HNS (*n* = 55)		
Big city > 100.000 inhabitants	8	14.5
Small town 15.000 bis 100.000 inhabitants	22	40.0
Rural area < 15.000 inhabitants	25	45.5
	Mean	SD
Number of employees *n* = 55	38.25	55.25

Characteristics of home nursing services with frequency (n) and valid percentage (%).

1 HNS in Germany have a provider that holds one of the following statuses: private/for-profit, non-profit (e.g. charitable and religious associations), or public (municipalities, municipal associations).

#### 3.3.4 Motivation to use INI in HNS

Assessment of nine motives for using INI (question: “What motivates you to use INI in HNS?”) was obtained using a 5-point Likert scale (5 = “I strongly agree”). The strongest motivators were: Belief in the efficacy of INI (M ± SD: 4.52 ± 0.750), Own basic nursing attitude (M ± SD: 4.50 ± 0.827), Enthusiasm for INI (M ± SD: 4.15 ± 1.089) and Positive experiences with INI in professional activities (M ± SD: 4.14 ± 1.014). See Table 3 for a complete overview.

**Table 3:** Motivation to use INI in HNS (scale 1-5).

	Mean	SD
Own basic nursing attitude (*n* = 20)	4.50	.827
Positive experiences with INI in professional activities (*n* = 21)	4.14	1.014
Key experience (*n* = 21)	3.71	1.347
Expanding the repertoire of nursing interventions (*n* = 19)	3.63	1.674
Enthusiasm for INI (*n* = 20)	4.15	1.089
Financial aspects (*n* = 18)	2.94	1.552
Retention of employees (*n* = 19)	3.05	1.615
Good placebo (*n* = 19)	2.00	1.333
Conviction of the effectiveness of INI (*n* = 21)	4.52	.750

#### 3.3.5 INI methods used in HNS, additional qualification, interest in training

Table 4 provides an overview of the methods of INI which are used by HNS, the additional qualifications of participants/nurses, and the number of participants who indicated an interest in training.

**Table 4:** INI methods used, additional qualification, further training.*

	INI used by HNS (*n* = 23)		Additional qualification of participants (*n* = 8)		Additional qualification of nursing staff (*n* = 9)		Participants interest for further training (*n* = 59)	
Methods INI	n	%	n	%	n	%	n	%
Acupressure	1	4.3	0	0	0	0	36	61.0
Anthrop. NC^a^	6	26.1	3	37.5	4	44.4	24	40.7
Aromatherapy	11	47.8	1	12.5	3	33.3	38	64.4
Compresses	13	56.5	2	25.0	5	55.5	35	59.3
Kneipp^b^	1	4.3	0	0	1	11.1	20	33.9
Rhythm.E.^c^	6	26.1	3	37.5	3	33.3	30	50.8
Inhalation	3	13.0	0	0	0	0	21	35.6
Reflexology	1	4.3	1	12.5	1	0	32	54.2
Tea^d^	14	60.9	0	0	2	22.2	27	45.8
Others	7^e^	30.4	3^f^	37.5	0	0	3^g^	5.1

INI methods provided, additional qualification, training needs with frequency (n) and valid percentage (%). *multiple choice.

a Anthroposophic nursing care.

b Kneipp-therapy.

c Rhythmical embrocation according to Wegmann/Hauschka.

d Medicinal herbal tea.

e e.g., hand massage, see Supplementary Table 1.

f Naturopathic practitioner, homeopathy, innerwise coach.

g e.g., seitai, Japanese method, embrocation.

The most commonly provided INI (question: “Which of the following methods do you use in your HNS?”) is herbal tea (60.9%, *n* = 14), the most frequently requested topic for training [question: “Of the INI mentioned, which one would you like to know more about (e.g., as part of a training course)”] is aromatherapy (64.4%, *n* = 38). For acupressure, the difference between use in home-care (4.3%, *n* = 1) and perceived need for training (61%, *n* = 36) is greatest.

Compresses used included ginger compresses on the chest or kidneys and curd compresses on the joints or chest. Herbal teas used included thyme internally for cough and thyme externally for MRSA disinfection. See Supplementary Table 1 for a complete overview of specific methods.

**Table 5:** Comparison INI user/ non-user.

Question	Total	INI user	Non-user
	Mean ± SD	Mean ± SD	Mean ± SD
Age	49.46 ± 11.91 (*n* = 56)	52.33 ± 10.032 (*n* = 21)	47.74 ± 12.733 (*n* = 35)
Time passed since passing nursing exam?	25.69 ± 12.73 (*n* = 54)	27.38 ± 11.042 (*n* = 21)	24.61 ± 13.756 (*n* = 33)
General attitude toward INI	8.37 ± 0.219 (*n* = 65)	9.34 ± 1.045 (*n* = 29)	7.58 ± 2.590 (*n* = 36)
Overall, how well do you feel informed about INI?^1^	5.39 ± 2.757 (*n* = 57)	7.05 ± 2.376 (*n* = 21)	4.42 ± 2.511 (*n* = 36)
How relevant were INI to your nursing education?^1^	3.41 ± 2.418 (*n* = 56)	4.38 ± 2.906 (*n* = 21)	2.83 ± 1.886 (*n* = 35)
I myself am explicitly asked about INI by patients.^2^	3.84 ± 1.192 (*n* = 57)	3.29 ± 1.347 (*n* = 21)	4.17 ± 0.971 (*n* = 36)
Counseling on INI is one of the tasks of nursing home-care.^2^	3.33 ± 1.215 (*n* = 57)	3.86 ± 1.062 (*n* = 21)	3.03 ± 1.207 (*n* = 36)
Application of INI is one of the tasks of nursing home-care^2^	3.44 ± 1.254 (*n* = 57)	4.05 ± 1.161 (*n* = 21)	3.08 ± 1.180 (*n* = 36)
I tend to take a positive view of my colleagues’ attitude toward INI.^2^	3.70 ± 1.077 (*n* = 56)	3.95 ± 1.024 (*n* = 21)	3.54 ± 1.094 (*n* = 35)
HNS that use INIs are particularly attractive to nurses.^2^	3.19 ± 1.217 (*n* = 57)	3.38 ± 1.161 (*n* = 21)	3.08 ± 1.251 (*n* = 36)
	Percentage		
Personal application (yes/no)	Yes: 86.4% (*n* = 57)	100% (*n* = 29)	76% (*n* = 28)
	No: 13.6% (*n* = 9)	–	24.3% (*n* = 9)
Nursing staff with additional qualification in INI (yes/no)	Yes: 15.8% (*n* = 9)	33.3% (*n* = 7)	5.6% (*n* = 2)
	No: 84.2% (*n* = 48)	66.7% (*n* = 14)	94.4% (*n* = 34)

SD, Standard deviation.

1 Scale 1–10.

2 Scale 1–5.

#### 3.3.6 Symptoms for which INI are used

Twenty nurses responded to the open-ended question for “For which diagnoses or nursing diagnoses do you use INI with your clients?” Two nurses stated that INI can be used for all types of health problems, depending on the nurses’ competencies and knowledge. The others (*n* = 18) reported a total of *n* = 51 health problems, which were grouped into 12 symptoms and nursing diagnoses. The results were pain (*n* = 8), respiratory problems (*n* = 6), anxiety/restlessness (*n* = 6), palliative care/oncology (*n* = 6), acute injury (*n* = 6), skin problems (*n* = 5), gastrointestinal problems (*n* = 6), depression/mood disorders (*n* = 4), cystitis (*n* = 2), sleep problems (*n* = 2), fever (*n* = 2), dementia (*n* = 1). See Supplementary Table 2 for a complete overview of symptoms.

#### 3.3.7 Reimbursement options

The multiple response question “What are the current options for reimbursement INI?” was answered by *n* = 59 participants. Approximately half (53%, *n* = 31) of the respondents indicated that they were not aware of any official reimbursement options. However, almost the same percentage said they were aware of options, specifically a privately paid service (59%, *n* = 35) and a fee-based service (9%, *n* = 5).

#### 3.3.8 Reasons for non-use (barriers)

Of the *n* = 37 nurses who do not use INI in the HNS, 76% (*n* = 28) use INI personally. The reasons given for not using INI in the HNS were “No way to bill for the service” (*n* = 26, 70.3%), “No staff with knowledge of INI” (*n* = 23, 62.2%), “No resources” (*n* = 7, 18.9%), “I do not believe in INI” (*n* = 2, 5.4%), and “other” (*n* = 7, 18.9%). The following five reasons were listed under “other”: “Little interest from staff,” “I’m not yet very familiar with INI,” “Consultation with doctors necessary, who often do not think much of INI,” “No doctor’s prescription,” “No demand from patients.”

#### 3.3.9 Relevance in nursing education

The relevance of INI in their nursing education (question: “How relevant were INI in your nursing education?”) was rated (by *n* = 56 participants) as rather low (M ± SD: 3.41 ± 2.418; scale: 1 = “not at all,” 10 = “very important”).

#### 3.3.10 Knowledge and need for information on INI

The personal level of knowledge about INI (question: “How well do you feel informed about INI overall?”) was assessed (by *n* = 57 participants) as rather average (M ± SD: 5.39 ± 2.757; scale: 1 = “very bad,” 10 = “very good”).

#### 3.3.11 Comparison between INI user and non-user

Comparison of responses from nurse managers of HNS using INI (users) and those that do not (non-user) reveals differences in several variables, as shown in Table 5.

#### 3.3.12 Challenges of using INI in HNS

The open-ended question regarding the challenges of INI in home nursing (“What challenges have you encountered in your experience with INI in HNS”) was answered by *n* = 45 participants. In the open-ended final question (“Do you have any comments about this study?”), *n* = 20 participants provided comments, three of which included remarks about opportunities and barriers. These three comments were included in the analysis. The answers were given in keywords and rarely in complete sentences, whereby the maximum number of characters was not used by any participant.

The data-driven content analysis revealed that not only challenges but also opportunities were mentioned. Seven major categories were identified: Financial aspects (*n* = 29 statements), Qualification (*n* = 17 statements), Limited resources (workforce and time; *n* = 12 statements), Patient and Family empowerment (*n* = 7 statements), Policy and health care system (*n* = 6 statements), Interprofessional collaboration (*n* = 5 statements), Patient interest (*n* = 4 statements). Both the challenges and the opportunities were very similar in content between nurse managers of HNS user (U) and HNS non-user (NU). All of the responses can be found in Supplementary Tables 3, 4.

### 3.4 Financial aspects

Most comments focused on financial aspects of INI (*n* = 29), with *n* = 25 expressing concern about the lack of reimbursement options. Four comments focused on patient financing. On one hand, there are patients who are willing to cover the costs (‘Costs (usually) willingly borne by patients when they feel the impact’ [U30]), while on the other hand, for many patients, it is not feasible to pay privately out of pocket (‘The question is almost always one of coverage, as clients already have high costs and do not want to incur additional costs, even for their own health.’ [U22]). The dilemma between perceived need for INI and the lack of funding leads to ‘sneaking’ services in funded services. [U21].

#### 3.4.1 Qualification

In second place (*n* = 17 comments) were various aspects of qualification, with the most common concern (*n* = 11 out of *n* = 17 statements) being a lack of knowledge about INI among staff. Further training was also discussed from a financial perspective, with one participant noting, “At the moment, systematic further training and education of nursing staff is too expensive and time-consuming” [NU36]. It was suggested that INI should be integrated into nursing education.

#### 3.4.2 Limited resources (staff and time)

In total, *n* = 12 comments were made about the time required for INI. While it was generally noted that there is not enough time, it was also acknowledged that INI require a significant amount of time.

#### 3.4.3 Patient and family caregiver education

There were *n* = 7 comments regarding the importance of patient and family caregiver education. These statements emphasized the need to provide information about the applications themselves and their mechanisms (“More information is needed to encourage people, especially about the potentially more complex nature and longer onset of effects” [U34]).

#### 3.4.4 Policy and health care system

*N* = 6 comments were attributed to policy and health care system. Physicians and health insurance companies should address the issue of INI and promote its implementation in HNS. Clarity was called for regarding the activities for which nurses are responsible (“Clear agreement on what can also be done without a doctor’s prescription” [NU32]).

#### 3.4.5 Interprofessional collaboration

Within the category of interprofessional collaboration (*n* = 5 comments), one aspect mentioned was the lack of acceptance of INI by physicians (“Few doctors are open to this kind of healing, dismissing it with the words: ‘If you believe in it’ “[U60]). The assumption of tasks by nurses was seen as a relief for doctors (“Also, in the context of the shortage of doctors, INI could provide some relief, as it can certainly accompany and manage some symptoms in the early stages of an illness” [NU47]).

#### 3.4.6 Patient interest

*N* = 4 comments were made regarding patient interest, two of which focused on the age of the patients (“A question of acceptance by the older population” [U22] and “Only patients who have had contact with INI in the past can be won over in old age” [U87]).

## 4 Discussion

To the best of our knowledge, this study provides the first quantitative and qualitative insights into the attitudes, knowledge, and use of INI among nurses working in HNS. With a response rate of only 5.1 percent, the results should be interpreted with caution. The low response rate is probably due to a lack of time (the resource ‘time’ is extremely scarce) and because research in the HNS setting is still very uncommon.

The general attitude of the participating nurses toward INI was clearly positive, with almost half of the them stating that they use INI in patient care. The most commonly addressed symptoms are pain, respiratory problems, anxiety, and palliative care. The main challenges reported are financial aspects, qualification and limited resources such as time and staff.

The overall positive attitude toward INI is consistent with the results of a survey conducted at German university hospitals by Hesmert et al., where midwifes and nurses expressed a higher favorability toward CIH compared to physicians and other healthcare professionals (15). In our study, the general attitude toward INI was more favorable among those participants who used INI in HNS compared to those who did not.

Motivators for using INI include the belief in the efficacy of INI, one’s own basic nursing attitude, enthusiasm for INI procedures, and positive experiences. This is consistent with data from a study conducted in Switzerland by Aveni et al. which found that personal experience is an important factor for healthcare professionals when forming their opinions and applying CIH (31).

The strong interest of nurses in INI aligns with the literature (57, 58). In our study, aromatherapy was the most frequently requested topic for further training, followed by acupressure. Similar, in the study of university hospitals by Hesmert et al. acupuncture/acupressure was the most frequently requested topic (15).

The need for systematic further education was clearly identified by the qualitative analysis of the survey. Lack of knowledge among staff and lack of training opportunities were identified as the second most common challenge regarding INI in HNS. Participants expressed that knowledge and skills in INI should be provided both during education and through increased opportunities for further training, seeing this as a prerequisite for the implementation of INI in HNS. Hall et al. also state that nurses still have very limited education in this area and lack professional frameworks to support them (50). Resources like the Integrative Nursing Handbook for Teachers in Nursing (INES) (59), and the Competency Catalog for Postgraduate Education in Medical Education developed by Valentini et al. (60) could address this need for qualification.

INI are used for a wide range of conditions, depending on nurses’ competencies and knowledge. The assessment of different indications is consistent with the literature, e.g., for pain (36). The specified INI also showed high concordance with those reported by patients as self-administered in a study conducted in primary care practices (9).

The lack of a specific billing code for direct reimbursement of INI suggests that professionals’ statements regarding its use are based on professional judgment rather than financial incentives. The survey results indicate that the INI can currently only be funded as a privately paid service. Funding was the most frequently cited challenge related to INI in HNS according to the analysis of the open-ended responses.

A potential advantage of INI in HNS is that they are often cost-effective and that INI applications can be learned by patients who can then apply them independently as directed by nurses. There is a reimbursement/billing code for nursing instructions. When nurses educate patients and family caregivers then this code could be used for billing purposes. The importance of patient and family caregiver education was supported by the qualitative analysis of the statements made in the survey responses in our study.

Interprofessional collaboration (IP) emerged as an issue as a result of the qualitative analysis of the survey. Despite a prevailing negative view among participants, discussions also highlighted opportunities supported by existing literature. Matthys et al. suggest that physician-nurse collaboration can positively impact several patient outcomes and pathologies, including hospital length of stay, blood pressure, and patient satisfaction. This is particularly relevant for patients with chronic conditions or those in need of longterm-care (61). Joos also considers close collaboration between nurses with additional qualification in INI and primary care physicians to be an important factor in the implementation of CIH and INI in patient care (62).

### 4.1 Limitations

The results should be interpreted with caution because of several important limitations. First, the questionnaire was not validated because, to our knowledge, this is the first study in this area in the German-speaking countries and no validated questionnaire has been published. Therefore, we needed to develop a new instrument. We discussed the questionnaire in our interprofessional team and research network and conducted pre-tests. Another limitation is the low response rate of 5.1%, which, however, is not uncommon in HNS research. Recent work in online monitoring of nursing staff in Baden-Württemberg, Germany, reported a response rate of 8.9% among HNS (63). However, a selection bias is highly probable. To mitigate this, the survey invitation email was worded neutrally. The structural characteristics of the providers of the realized sample closely matched the provider structure of the entire population of nursing facilities in Germany (64).

It is possible that the survey link was distributed beyond the intended list of HNS, as there was no personalized access. While multiple participation by participants was generally possible, it was considered unlikely given the need to provide false demographic information.

Lastly, qualitative data were collected exclusively through two open-ended questions which usually lead to short, condensed responses. More complex content which might emerge in an interview was likely missed.

## 5 Conclusion

By exploring the challenges and factors that facilitate or hinder the use of INI, this study provides a broad overview and a starting point for further research. Insights into the perspective of nurses working in HNS on INI in their work context were gained. This should be explored further through qualitative interviews. With demographic changes leading to an increase in the number of people needing care, it is increasingly important to empower patients for self-care. INI are good, low-threshold, and safe methods for self-care. Therefore, it is important that they are taught in nursing education and training, that they are reimbursed, and that they are used in an interprofessional primary care setting. The structures within the German healthcare system urgently need to be adapted in order to initiate this development.

## Data Availability

The raw data supporting the conclusions of this article will be made available by the authors, without undue reservation.
