# Supplementary material for: Integrative nursing interventions: knowledge, attitudes and practice in home nursing services in Germany—a quantitative and qualitative online survey
Source: Front Med (Lausanne). 2024 Oct 3;11:1438035. doi: 10.3389/fmed.2024.1438035 (PMC11484039; doi:10.3389/fmed.2024.1438035)
Supplement: Supplementary file 1 [file Data_Sheet_1.docx]

Supplementary Datasheet 1

Table 1 Specific methods (Question 2.11 anwers to methods “namely” and “others”)

| **Answers** | **Participant number*** |
| --- | --- |
| **namely** |  |
| Rhythmische Einreibungen, Wickel und Auflagen  ***Rhythmic embrocation, compresses and wraps*** | U21 |
| Einreibungen Auflagen pflegerische Gesten  ***Embrocations, compresses, Rolf Heine's nursing gestures*** | U22 |
| vor allem Rh Einreibungen, aber auch Wickel und Auflagen, Tees, Substanzen, pflegerische Haltung (z.B. pflegerische. Gesten nach Rolf Heine)  ***Especially rhythmic embrocation, but also wraps and compresses, teas, substances, nursing gestures (e.g. Rolf Heine's nursing gestures).*** | U30 |
| Rhythmische Einreibungen, Öldispersionsbäder, Wickel und Auflagen, Einreibungen, Klingende Waschung  ***Rhythmical embrocation, oil dispersion baths, compresses and wraps, compresses, sound washes*** | U41 |
| klingendes Fußbad  ***Sound foot bath*** | U67 |
| Wickel, Auflagen, Rhythmische Einreibungen nach Wegmann Hauschka, Klingende Waschungen, Inhalationen mit ätherischen Ölen, Fußbäder  ***wraps, compresses, rhythmic Wegmann Hauschka compresses, sound washes, inhalations with essential oils, foot baths*** | U89 |
| Einreibungen, Duftsticks, Inhalation, Massagen, Bäder  ***Embrocations, aroma sticks, inhalation, massages, baths*** | U16 |
| Als Öle für Einreibungen, Massageöle mit ätherischen Ölen (z.B. Lavendelöl)  ***Essential oils for embrocation (e.g. lavender)*** | U21 |
| gelegentlich bei unruhigen Menschen als Duftlampe oder Duftkissen  ***Occasionally as an aroma lamp or aroma pillow for restless people*** | U25 |
| Raumbeduftung und Einreibungen  ***Room scenting and embrocations*** | U58 |
| Düfte  ***Essential oils*** | U66 |
| Brustwicklel, Bauchwickel  ***Chest compress, stomach compress*** | U16 |
| Ingwerwickel Niere, Brust), Senfmehlwickel, Brust), Zitronen-Salz-Wickel (Brust), Öl-Wickel (Brust), Öldispersionsbäder, Quarkwickel (Brust, Gelenke), Retterspitz (Extremitäten), Schafgarbenleberwickel und was sonst noch verordnet/gewünscht wird  ***Ingwer wraps (kidneys, chest), Senfmeh wraps (chest), lemon-salt wraps (chest), oil wraps (chest), oil dispersion baths, curd wraps (chest, joints), rescuer's tip (extremities), yarrow liver wraps and whatever else is prescribed/requested***. | U21 |
| diverse, z.B. Blasen-, Brust-, Gelenk- Auflagen  ***Various, e.g. bladder, chest, joint wraps*** | U30 |
| Senfmehl, Lavendelölläppchen, Quark  ***Mustard flour, lavender oil sachets, curd*** | U41 |
| vor allem Wärmewickel  ***especially warm compresses*** | U87 |
| Senf, Ingwer, Quark, Salbenauflagen, Ölauflagen  ***mustard, ginger, quark, ointments, oil packs*** | U89 |
| Dampfinhalation mit z. B. Kamille, PARI-Boy Inhalation  ***Steam inhalation with e.g. chamomile, PARI-Boy inhalation*** | U21 |
| Salbei, Kamille  ***Sage, chamomile*** | U41 |
| Thymian,Lavendel, Eucalyptus, Rose (Duftlampe)  ***Thyme, lavender, eucalyptus, rose (aroma lamp)*** | U89 |
| Thymian, Salbei  ***Thyme, Sage*** | U16 |
| Je nach Verordnung oder Wunsch (Equisetum, Kamille, Thymian, Cystus, Ingwer, Rosmarin, Lavendel u.v.m.)  ***As prescribed or requested (equisetum, camomile, thyme, cystus, ginger, rosemary, lavender, etc.)*** | U21 |
| je nach Bedarf  ***according to need*** | U22 |
| bei unruhigen Menschen oder auch zur besseren Verdauung  ***for restless people or for better digestion*** | U25 |
| z.B, Thymiantee f. MRSA-Sanierung äußerlich, Nieren-Blasentee, Hustentee, Schafgarbentee u.a.m.  ***e.g. thymiantee for external MRSA disinfection, nephritic tea, cough tea, yarrowtee, etc***. | U30 |
| Schafgarbe, Kamille, Wermut, u. v. m.  ***yarrow, chamomile, courage, etc***. | U41 |
| evidenzbasierte Heilkräuter wie z.B. Thymian bei Husten usw  ***Evidence-based herbs such as thyme for coughs, etc***. | U58 |
| Nieren- und Blasentee, Beruhigungstee  ***Kidney and bladder tea, calming tea*** | U60 |
| Calendula, Schachtelhalm, Brennessel, Thymian, Lavendel, Rosmarin,  ***Calendula, marigold, feverfew, thyme, lavender, rosemary***, | U89 |
| **Others** |  |
| Handmassage  ***Hand massage*** | U24 |
| Wickel,  ***wraps*** | U34 |
| Einreibungen mit ätherischen Ölen, z. B. Aconit, Solum, Lavendel u. v. m. , Öldispersionsbäder  ***embrocation with essential oils, e.g., aconit, solum, lavender, etc., oil dispersion baths***. | U41 |
| Kohle tabletten  ***Coal tablets*** | U60 |
| Wala Schmerzöl  ***Wala Pain Oil*** | U67 |
| Öle zur Einreibung, palliativ, Öle zur Behandlung von Rötungen/ intertrigo  ***Essential oils for embrocation, palliative, oil for the treatment of redness / intertrigo*** | U72 |
| Anwendung Naturheilkundlicher Salben je nach Patientenwunsch  ***Application of naturopathic ointments according to patient's wishes*** | U76 |

Note: *U=user, NU=non-user

Table 2 Symptoms for which INI are used

| **Answers** | **Participant number*** |
| --- | --- |
| bei allen Diagnosen gibt es Anwendungsmöglichkeiten.  ***There are application possibilities for all diagnoses*** | U30 |
| Bei sämtlichen Diagnosen und Erkrankungen, je nach Wissenstand und genaue Kenntnis der Anwendung  ***For all diagnoses and diseases, depending on the level of knowledge and exact knowledge of the application.*** | U72 |
| Bronchitis, Lungenentzündung, entzündete, schmerzende Gelenke, Blutergüsse, Tumorerkrankungen  ***Bronchitis, lung inflammation, inflamed, painful joints, bruising, tumors*** | U21 |
| Bronchitis, steifer Nacken, Durchfall, Prellungen  ***Bronchitis, stiff neck, diarrhea, bruises*** | U87 |
| Demenz, Schlaganfall  ***Dementia, Stroke*** | U24 |
| Die Therapie wird erst nach Anweisung vom Arzt angewendet.  ***The therapy is administered only as directed by the physician.*** | U52 |
| Demenz und Unruhe, Magen-Darmproblemen  ***Dementia and agitation, gastrointestinal problems*** | U25 |
| Einschschlafprobleme, Unruhe, Leichtes Fieber, Erkältungssymptome, Stimmungsschwankungen  ***Insomnia, restlessness, low-grade fever, cold symptoms, mood swings*** | U58 |
| Fieber, Pilzerkrankung,Erkältung, Verstimmungen, Magen – Darmerkrankung  ***Fever, fungal disease, cold, indigestion, stomach and intestinal disorders*** | U34 |
| Herpes Zoster, Unruhe  ***Herpes zoster, restlessness*** | U67 |
| Intertrigo, Schmerzzustände, chronische Schmerzen,  ***Intertrigo, painful conditions, chronic pain,*** | U55 |
| Meist bei Hautproblemen, Schmerzen oder Hämatomen  ***Most commonly used for skin problems, pain or bruising.*** | U76 |
| Nieren- und Blasentee bei Patienten mit starker neigung zur Kronischen Blasenentzündung als Dauertherapie. Beruhigungstee bei Personen mit leichter Unruhe. Kohle Tabletten bei Magenverstimmung, Durchfall oder Verstopfung Vergiftungserscheinungen  ***Kidney and bladder tea for patients with a strong tendency to cystitis as a long-term therapy. Calming tea for people with mild anxiety. Coal tablets for indigestion, diarrhea or constipation.*** | U60 |
| Paliativsituation, diverse Hautkrankheiten (Ausschlag, Herpes Zoster, Altershaut, Juckreitz) Erysipel, Beinödeme, Erkältungen, Blasenentzündungen, Pneumonie, Obstipation, Rheumatologische Erkrankungen....  ***Palliative situation, various skin diseases (rashes, herpes zoster, aging skin, itching), erysipelas, leg ulcers, colds, bladder infections, pneumonia, constipation, rheumatologic diseases ....*** | U89 |
| Palliative Situationen Gelenkbeschwerden Leberbeschwerden Kardiale Symptomatik Innere Unruhe  ***Palliative Situations Joint Diseases Liver Diseases Cardiac Symptoms Internal Unrest*** | U22 |
| Palliativversorgung  ***Palliative Care*** | U77 |
| Schlafstörungen, Ängste  ***Sleep disorders, anxiety*** | U66 |
| Schwellungen, Entzündungen  ***Swelling, inflammation*** | U63 |
| unkomplizierte Erkältungskrankheiten der oberen Atemwege, oberflächliche Beinvenenthrombose, Hämatome, Schmerzen, Angstzustände, Depressionen, im Rahmen der Sterbepflege  ***uncomplicated upper respiratory tract infections, superficial leg vein thrombosis, hematoma, pain, anxiety, depression, hospice care*** | U41 |
| vowiegend Krebserkrankungen, Hausapotheke  ***cancer, home pharmacy*** | U16 |

Note: *U=user, NU=non-user

Table 3 Challenges

| **Answers** | **Participant number*** |
| --- | --- |
| - sollte in die Ausbildung mit aufgenommen werden - klare Absprachen/ vereinbarungen was auch ohne ärztliche Anordnung durchgeführt werden kann  ***should be included in the nursing education - Clear agreement on what can also be done without a doctor's prescription.*** | NU32 |
| -Wenig Wissen -Abrechnungsmöglichkeiten -Wenig Interesse seitens Kunde  ***Lack of knowledge - Lack of reimbursement - Lack of customer interest*** | NU51 |
| Abrechnen der Leistungen  ***Reimbursement of services*** | NU43 |
| Abrechnung  ***Reimbursement*** | NU44 |
| Abrechnung der Leistung, Haftung  ***Reimbursement, Liability*** | NU71 |
| Abrechnungspakete -> Abrechnung über Kasse, nicht Privat Schulung der Mitarbeiter  ***Billing Packages -> reimbursement via health insurance, not private; Training of Employees*** | NU27 |
| Annahme und Aufklärung über die Wirksamkeit der Kunden, da viele skeptisch sind  ***Acceptance and education of patients about efficacy, as many are skeptical*** | NU86 |
| Ärzte und Krankenkassen, die da nicht mitziehen und vor allem die Pharmaindustrie!  ***Doctors and health insurance companies that do not go along with this, and especially the pharmaceutical industry!*** | U67 |
| ausreichendes Wissen fehlt, Patienten sind nicht bereit Leistungen privat zu zahlen  ***Lack of knowledge, patients unwilling to pay for services privately*** | NU73 |
| Dass sie nicht als Leistungsmodule finanziert sind. Viele Menschen können sich keinen täglichen Wickel für, je nach Zeitaufwand, z.B. 35 Euro leisten. Oder die Pflegedienste müssen die Leistungen irgendwo unter die Pflege mogeln  ***That they are not financed as service modules. Many people cannot afford a daily wrap/compress for 35 euros,*** ***depending on the amount of time required. Or the nursing services have to sneak the service in somewhere under nursing.*** | U21 |
| den Klienten und auch deren Angehörige von den positiven Eigenschaften der NP zu überzeugen. Vielen ist immer noch nicht klar, dass die NP positiven Einfluss auf Körper, Geist und Wohlbefinden haben.  ***Convincing clients and their families of the positive qualities of NNI. Many people are still unaware of the positive effects of NNI on body, mind and well-being.*** | NU85 |
| Die Abrechnung mit der Krankenkasse  ***Billing the health insurance company*** | NU40 |
| Die Finanzierbarkeit und die zusätzliche Zeit.  ***Financing and additional time.*** | NU35 |
| Die Kunden versuchen statt Antibiotika Tee zu trinken. Keine gute Idee  ***Patients try to drink tea instead of antibiotics. Not a good idea*** | NU56 |
| Die Nichtfinanzierung der NP über Verordnung häusliche Pflege, Schulungsangebote zum Thema und Sensibilisierung der MA für das Thema  ***Non-funding of NNI through home care regulations, training on the topic, and sensitization of staff to the topic.*** | U25 |
| Die vorhandenen Zeitressourcen - es kann derzeit nicht adäquat und kostendeckend abgerechnet werden  ***Existing time resources - cannot currently be billed appropriately and cost-covering*** | NU68 |
| Eine Frage der Akzeptanz bei der älteren Bevölkerung . Die Frage ist fast immer die Kostenübernahme ,da die Klienten sowieso meist schon hohe Kosten haben wollen sie nicht noch zusätzliche Kosten haben .Auch nicht für ihre eigene Gesundheit.  ***A question of acceptance by the older population. The question is almost always one of coverage, as clients already have high costs and do not want to incur additional costs, even for their own health.***. | U22 |
| Es gilt, die Pflegeffach- und Betreuungskräfte zu schulen, Begeisterung und Erkennen des Mehrwerts für NP auszulösen, Kontinuität der Durchführung.  ***It is a matter of educating the nursing and support staff, generating enthusiasm and recognition of the benefits of NNI, and ensuring continuity of implementation.*** | NU26 |
| Es muss mehr informiert werden um die Menschen dazu zu bringen. Vorallem das es etwas aufwenduger sein kann und die Wirkungsweise länger dauern kann  ***More information is needed to encourage people, especially about the potentially more complex nature and longer onset of effects.*** | U34 |
| Es wird von den meisten Angehörigen und zu Pflegenden mit skepsis angenommen, aber nach einer Zeit gut akzeptiert und angewendet. Die wenigsten Ärzte sind offen für diese Art der Heilkunde und tun diese gerne mit den Worten ab: wenn Sie daran glauben  ***It is received with skepticism by most relatives and caregivers, but after a time it is well accepted and applied. 'Few doctors are open to this kind of healing, dismissing it with the words: 'If you believe in it.'*** | U60 |
| Fehlende Finanzierung durch die Kranken- bzw. Pflegekassen  ***Lack of funding from health or long-term care insurers*** | U76 |
| Fehlende Kenntnisse über NP Methoden Einschränkungen im Wohnumfeld der Patienten  ***Lack of knowledge about NNI methods Limitations of the patient's home environment*** | NU84 |
| Habe mich noch nicht mit dem Thema befasst  ***Not yet familiar with the topic*** | NU78 |
| Hohe Kosten, keine oder nur geringe Kosten übernehme seitens der Kassen, zu wenig Fortbildung zu diesem Thema, so das alle Mitarbeiter informiert sind  ***High costs, little or no reimbursement from health plans, too little training on this topic to keep all employees informed.*** | U72 |
| Im Moment ist eine systematische Fortbildung und Schulung der Pflegekräfte zu teuer und aufwendig  ***At the moment, systematic further training and education of nursing staff is too expensive and time-consuming.*** | NU36 |
| keine Abrechnung möglich, schwierig bei der Umsetzung von NP  ***No billing possible, difficult to implement NNI*** | NU57 |
| Keine Leistung der Häuslichen Krankenpflege so dass nicht mit der KK abgerechnet werden kann als Leistungspaket bei Pflegebedürftigkeit und abrechnung über die Pflegekasse ebenfalls nicht definiert. Fraglich ist, ob diese Leistung als Leistungspaket  ***Not a service of home health care, so that it cannot be billed to the health insurance company as a package of services in the event of need for long-term care, and billing via the health insurance company is also not defined. It is questionable whether this service as a service package*** | NU83 |
| keine Refinanzierung, hoher zeitl Aufwand, Fortbildungsanerkennung  ***No re-funding, high time commitment, training recognition*** | U58 |
| keine Zeit dafür  ***No time for it*** | NU45 |
| keine Zeit zu wenig Mitarbeiter  ***No time, too few staff*** | U24 |
| Kosten werden nicht von der Kasse übernommen  ***Costs not covered by insurance*** | U55 |
| Leistungsabrechnung  ***Reimbursement*** | U66 |
| Leistungskatalog und geeignete Weiterbildungen in dem Bereich  ***Service catalog and appropriate training in the area*** | NU47 |
| Mehr Bekanntheit an Naturheilkunde Veröffentlichen!!  ***More popularity for naturopathy, publish!*** | U88 |
| Nur Klienten, die früher Kontakt mit NP hatten, sind auch im Alter dafür zu gewinnen. Jüngere Klienten, die schon einmal gute Erfahrungen damit gemacht haben, möchten dies auch gerne in der Pflege anwenden  ***Only patients who have had contact with NNI in the past can be won over in old age. Younger clients who have had good experiences with NNI want to use it in their care.*** | U87 |
| Ohne ärztliche Zustimmung wird es als Einmischung verstanden Globuli sind umstritten  ***Without a physician's approval, it is considered interference Globuli are controversial.*** | NU42 |
| Umgang mit den allopathisch orientierten Hausarztpraxen. Zeitliche Resourcen  ***Dealing with allopathic family practices. Time Resources*** | U89 |
| Vergütung der Leistung für entsprechende Zeit  ***Pay for performance over time*** | NU46 |
| Wie gelingt es die Selbstheilungskräfte des Menschen zu stimulieren, so dass Freude am und Verantwortung für den eigenen Körper für ihn wieder erlebbar ist, wenn eine Phase der Kränkung und des Krankwerdens diese eigene Kraft schwinden ließ.  ***How is it possible to stimulate the self-healing powers of the human being, so that joy and responsibility for one's own body can be experienced again, when a phase of injury and illness has made one's own powers dwindle.*** | U41 |
| Zeitbedarf, Kosten (wird i.d.R. von Klienten gern getragen, wenn sie die Wirkung spüren); Zeit- und Finanzbedarf f. Fortbildungen f. Mitarbeiter  ***Time commitment, costs (usually willingly borne by patients when they feel the impact); time and money required for staff training.*** | U30 |
| Zeitlicher Aspekt und Grundwissen der Mitarbeiter  ***Time aspect and the basic knowledge of the staff*** | NU74 |
| Zeitlicher Faktor, Qualifikationsmöglichkeiten, Befürwortung durch GF und Vorstand, Abrechnungsmöglichkeiten  ***Time factor, Qualification possibilities, Approval by managing director and board, Billing possibilities*** | U77 |
| Zu geringe Kenntnisse, keine Abrechnungsmöglichkeiten, keine personellen Ressourcen  ***Too Little Knowledge, No Billing Options, No Human Resources*** | NU31 |
| Zu wenig Kenntnis bei Klienten und Mitarbeitern über Sinn und Zweck. wie sollen die Leistungen abgerechnet werden?  ***Too little knowledge among patients and staff about the purpose of the service and how it should be billed.*** | NU53 |
| Zu wenig Zeit, Aufwand wird nicht vergütet  ***Too little time, effort not compensated*** | U63 |

Note: *U=user, NU=non-user

Table 4 Comments

| **Answers** | **Participant number*** |
| --- | --- |
| Die Kassen sowie die Ärzte sollten mehr in dieses Thema integriert werden. Wir haben gesehen was zur Corona Pandemie passiert ist. Es hätte sicher Naturheilpraktische Möglichkeiten gegeben ... diese wurden nicht nur ignoriert, diese wurden sogar blockiert [47]  ***The insurance companies as well as the doctors should be more involved in this issue. We have seen what happened with the Corona pandemic. There were certainly naturopathic options ... these were not only ignored, they were even blocked***. | U55 |
| Eine tolle Fragestellung und eine klasse Initiative  ***A great question and a great initiative!*** | U21 |
| Finde ich toll, dass es mal einen neuen Ansatz gibt.  ***I think it is great that there is a new approach.*** | NU42 |
| Ich begrüße Ihre Initiative zu diesem Thema sehr und freue mich über diesen Fokus, da er mir aus dem HERZEN spricht  ***I very much welcome your initiative on this subject and am happy about this focus, because it speaks to my heart.*** | U89 |
| Ich bin überzeugt das einige NP eine Krankheitsbild verbessern können und Leid vermindern. Auch im Zuge des Ärztemangels könnte man mit NP ein wenig Entlastung bringen, da man einiges bestimmt im Anfangsstadium einer Erkrankung begleiten kann  ***I am convinced that some NNI can improve a disease and reduce suffering. Also, in the context of the shortage of doctors, NNI could provide some relief, as it can certainly accompany and manage some symptoms in the early stages of an illness.*** | NU47 |
| Ich freue mich, dass sie unternommen wird!  ***I am glad that it is being done!*** | U41 |
| Ich habe eine kleine Tagespflege 7/9 Tagesgäste  ***I have a small day care center 7/9 Day guests*** | U88 |
| Im Moment nicht  ***Not at the moment*** | U34 |
| interessant wäre vor allem die naturheilkundliche Schmerztherapie  ***Of particular interest is naturopathic pain therapy.*** | NU53 |
| ***Nein***  ***No*** | NU31, NU26, NU27, NU71, NU78 |
| Nein  ***No*** | U66, NU74 |
| Super Idee!  ***Great idea!*** | NU68 |
| Toll das so etwas mal durchgeführt wird . Ich wäre sehr am Ergebnis interessiert.  ***Great that something like this is being done. I would be very interested in the results.*** | U22 |
| Wahrscheinlich genau so unnützlich wie die Anderen Studien  ***Probably just as useless as the other studies*** | NU56 |

Note: *U=user, NU=non-user

**Questionnaire German-English**


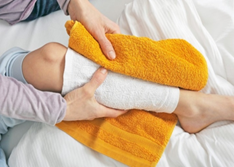


**
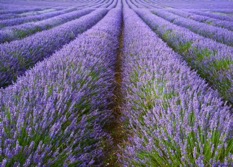
**

** Institut für Allgemeinmedizin und Interprofessionelle Versorgung**

**Naturheilkundliche Pflege und Hausmittel –**

**eine Online-Befragung ambulanter Pflegedienste in Baden-Württemberg**

**Integrative nursing interventions and home remedies –**

**An online survey of home nursing services in Baden-Württemberg**

**Wer kann bei der Befragung mitmachen?**Die Befragung richtet sich an Leitungspersonen **aller ambulanten Pflegedienste in Baden-Württemberg**

**Who is eligible to take part in the survey?**

The survey targets nursing managers **of all home nursing services in Baden-Württemberg**

**Worum geht es in der Befragung?**
**Naturheilkundliche Pflege und Hausmittel (NP)** (z. B. Wickel, Auflagen, Aromapflege, Waschungen, Akupressur etc. gelten als wichtige Ressourcen in der Gesunderhaltung der Bevölkerung sowie in der Prävention und Therapie von Krankheiten, für deren Wirksamkeit zunehmend Erkenntnisse aus systematischer Forschung vorliegen. Es sind zum Teil einfache Maßnahmen, die Patient/-innen nach Anleitung selbst durchführen können. Bei komplexen Maßnahmen oder pflegebedürftigen Patient/-innen kann die Übernahme durch Pflegefachpersonen notwendig werden.

**What is the survey about?**

Integrative nursing interventions and Home Remedies (INI) (e.g., compresses, aromatherapy, ablutions, acupressure, etc.) are considered important resources in maintaining the health of the population and in preventing and treating disease, and there is increasing evidence of their effectiveness from systematic research. Some of these are low-threshold interventions that patients can perform themselves with instructions. In the case of complex interventions or patients in need of care, it may be necessary for nurses to take over.

Obwohl bekannt ist, dass zahlreiche ambulante Pflegedienste naturheilkundliche Pflege durchführen, fehlen Daten dazu, in welchem Umfang dies angeboten wird und welche Kenntnisse vorhanden sind. Mit Ihrer Beteiligung an der Umfrage können Sie dazu beitragen, naturheilkundliche Pflegemaßnahmen in den Leistungskatalog der Pflegedienste aufzunehmen. Außerdem können auf Basis der Umfrageergebnisse gezielt Fortbildungen entwickelt und angeboten werden.

Although it is known that many home nursing services provide integrative nursing interventions, there is a lack of data on the extent to which this is provided and the knowledge that is available. By participating in the survey, you can help ensure that integrative nursing interventions are included in the funded services offered by home nursing services. The results of the survey can also be used to develop and offer targeted training programs.

Mit der vorliegenden Befragung möchten wir **Ihre Erfahrungen, Meinungen und Ihren Informationsbedarf** zu NP erfahren.
Für die Beantwortung des Fragebogens brauchen Sie ca. **5- 10 min.**

Für die Teilnahme an der Umfrage bedanken wir uns mit einer kostenlosen Online-Fortbildung (90 Minuten) für Ihre Mitarbeitenden zum Thema

*„Naturheilkundliche Pflegemaßnahmen in der ambulanten Pflege erfolgreich anwenden“*

*The purpose of this survey is to learn about* **your experiences, attitudes, and information needs** *regarding INI.*

*It will take you approximately* **5-10 minutes** *to complete the questionnaire.*

*We would like to thank you for your participation in the survey with a free 90-minute online training course for your employees on the following topic*

*"Successfully applying integrative nursing interventions in home nursing"*

**Wer führt die Befragungsstudie durch?**
Die Studie wird im Rahmen **einer pflegewissenschaftlichen Doktorarbeit am Institut für Allgemeinmedizin, Tübingen unter Leitung von Prof. Dr. Stefanie Joos durchgeführt.**

**Who is conducting the survey?**

The study is part of a **doctoral thesis in nursing science at the Institute of General Medicine in Tübingen, Germany, under the direction of Prof. Dr. Stefanie Joos.**

**Was geschieht mit Ihren Angaben?**Die Daten werden vollständig anonym erhoben, sodass ein Rückschluss auf Ihre Person nicht möglich ist. Es werden keine IP-Adressen aufgezeichnet und keine Cookies verwendet, wodurch ein Zwischenspeichern leider nicht möglich ist. Mit dem Ausfüllen dieses Fragebogens erklären Sie sich damit einverstanden, dass die anonym erhobenen Daten ausgewertet und veröffentlicht werden dürfen.

Die Aufbewahrung und Auswertung der Daten erfolgt im Rahmen der gesetzlichen Datenschutzbestimmungen.

**What will happen to your data?**

The data is collected anonymously, so it is not possible to identify you personally. No IP addresses are recorded and no cookies are used, it is unfortunately not possible to store the data temporarily. By completing this questionnaire, you agree that the anonymous data collected may be analyzed and published.

The data will be stored and analyzed in accordance with data protection regulations.

**Sie haben noch Fragen?**Dann wenden Sie sich gerne an uns.

Regina Stolz M.A. Pflegewissenschaft, Krankenschwester ([regina.stolz@med.uni-tuebingen.de](mailto:regina.stolz@med.uni-tuebingen.de))

Prof. Dr. med. Stefanie Joos, Ärztliche Direktorin, Institut für Allgemeinmedizin und Interprofessionelle Versorgung, Tübingen ([stefanie.joos@med.uni-tuebingen.de](mailto:stefanie.joos@med.uni-tuebingen.de))

**Do you have any questions?**

Please feel free to contact us.

Regina Stolz M.A. Nursing Science, RN (regina.stolz@med.uni-tuebingen.de)

Prof. Dr Stefanie Joos, Medical Director, Institute of General Practice and Interprofessional Care, Tübingen (stefanie.joos@med.uni-tuebingen.de)

**Herzlichen Dank…**
… schon an dieser Stelle für Ihre Unterstützung!

Die unverbindliche Anmeldung zur Online-Fortbildung ist nach Abschluss der Befragung möglich. Sie lässt keinen Rückschluss auf Ihre Angaben in der Befragung zu.

**Thank you very much...**

... for your support at this point!

After completing the survey, you will be able to register for the online training program without obligation. It does not allow any conclusions to be drawn about the information you provide in the survey.

## 1. Einstellungen

## 1. Attitudes

## 1.1 Wie ist Ihre Grundeinstellung zu naturheilkundlicher Pflege (NP)?

## 1.1 What is your general attitude towards integrative nursing interventions (INI)?

## Bitte stellen Sie mit dem Schieberegler den entsprechenden Wert ein.

## *(Please use the slider to set the appropriate value.)*

## .

## „Beispiele für Naturheilkundliche und komplementäre Pflege:

## „Examples for integrative and complementary nursing Interventions:

## Akupressur, Anthroposophische Pflege, Aromapflege, Fußbäder Heilpflanzen-Tee, Inhalationen, Kneipp`sche Anwendungen, Leberwickel, Quarkwickel, rhythmische Einreibungen nach Wegmann/Hauschka, Wechselduschen

## Acupressure, anthroposophic care, aromatherapie, foot baths, medicinal plant teas, inhalations, Kneipp treatments, liver compress, curd compresses, rhythmical embrocations according to Wegmann/Hauschka, alternating showers

| sehrablehnendvery |  | sehrbefürwortendvery |
| --- | --- | --- |
| unfavorable |  | favorable |

## |______|______|______|______|______|______|______|______|______|

## 1.2 Wenden Sie für sich selbst oder in ihrem privaten Umfeld NP an?

## 1.2 Do you use INI personally?

## Bitte wählen Sie die zutreffende Antwort aus.

## Please select the appropriate answer.

|  | ja  yes |
| --- | --- |
|  | nein  no |

## 2. Versorgungsrealität naturheilkundliche Pflege im ambulanten Pflegedienst

## 2. Integrative Nursing Interventions in the Home nursing setting

## 2.1 Wenden Sie NP in Ihrem Pflegedienst an?

## 2.1 Do you use INI in home nursing service?

## Bitte wählen Sie die zutreffende Antwort aus.

## Please select the appropriate answer.

|  | ja  yes |
| --- | --- |
|  | nein  no |

## 2.1.1 Wenn ja, welche der folgenden Methoden?

## 2.1.1 If so, which of the following methods?

*Bitte geben Sie* ***alle*** *zutreffenden Methoden an.*

*Please indicate* ***all*** *applicable methods.*

| Akupressur  Acupressure |  |
| --- | --- |
| Anthroposophische Pflege und zwar  Anthroposophic nursing care, namely |  |
| Aromapflege und zwar  Aromatherapy, namely |  |
| *Wickel und Auflagen und zwar*  Compresses, namely |  |
| *Kneipp`sche Anwendungen (Wechselbäder, Güsse)*  *Kneipp-therapy (contrast baths, watering)* |  |
| Rhythmische Einreibungen nach Wegmann/ Hauschka  Rhytmical embrocation according to Wegmann/ Hauschka |  |
| *Inhalationen und zwar*  *Inhalation, namely* |  |
| Reflexzonenmassage  Reflexology |  |
| Heilpflanzentee und zwar  Medicinal herbal tea, namely |  |
| Andere: _________________________  Other: _________________________ |  |

### 2.1.2 Wenn ja, bei welchen Diagnosen oder Pflegediagnosen wenden Sie NP bei Ihren Klient/-innen an?

**2.1.2 If so, for which diagnoses or nursing diagnoses do you use INI with your clients?**

______________________________________________________________________________________________________________________________________________________________________________________________________________________________

## 2.1.3 Wenn ja, Was motiviert Sie, NP in Ihrem Pflegedienst anzuwenden?

## 2.1.3 If so, what motivates you to use INI in home nursing service?

## *Bitte wählen Sie alle zutreffenden Antworten aus.*

## *Please select all appropriate answers.*

|  | Stimme überhaupt nicht zu  Strongly disagree |  |  |  | Stimme voll und ganz zu  Strongly agree |
| --- | --- | --- | --- | --- | --- |
| Eigene pflegerische Grundhaltung  Own basic nursing attitude |  |  |  |  |  |
| Positive Erfahrungen mit NP in der beruflichen Tätigkeit.  Positive experiences with INI in professional activities. |  |  |  |  |  |
| Schlüsselerlebnis (eigenes oder in Familie-/Freundeskreis)  Key experience (own or with family/friends) |  |  |  |  |  |
| Erweiterung des Pflegespektrums  Expanding the repertoire of nursing interventions |  |  |  |  |  |
| Begeisterung für NP  Enthusiasm for INI |  |  |  |  |  |
| Finanzielle Aspekte  Financial aspects |  |  |  |  |  |
| Bindung von Mitarbeitenden  Retention of employees |  |  |  |  |  |
| Gutes Placebo  Good placebo |  |  |  |  |  |
| Überzeugung von der Wirksamkeit von NP  Conviction of the effectiveness of INI |  |  |  |  |  |

## 2.1.4 Wenn nein, aus welchen Gründen?

## 2.1.4 If no, what are the reasons?

## *Bitte wählen Sie alle zutreffenden Antworten aus.*

## *Please select all appropriate answers.*

|  | Keine Ressourcen  No resources |
| --- | --- |
|  | Keine Möglichkeit die Leistung abzurechen  No possibility to bill for the service |
|  | Ich halte nichts von NP  I don’t believe in INI |
|  | Kein Personal mit Kenntnissen in NP  No staff with knowledge on INI |
|  | Sonstiges und zwar  Others, namely |

## 2.2 Wie häufig fragen Klient/-innen und Angehörige nach NP?

## 2.2 How often do clients and relatives ask about INI?

## *Bitte wählen Sie in jeder Zeile eine Antwort aus.*

## *Please select one answer in each line.*

|  | Sehr häufig (mehrmals pro Woche)  Very often (several times a week) | Häufig (ca. 1x pro Woche)  Frequently (approx. 1x per week) | Gelegentlich (ca. 1x pro Quartal)  Occasionally (approx. 1x per quarter) | Selten (ca. 1x pro Halbjahr)  Rarely (approx. once every six months) | nie  never |
| --- | --- | --- | --- | --- | --- |
| Ich selbst werde von Klient/-innen explizit zu NP gefragt.  I myself am explicitly asked about INI by clients. |  |  |  |  |  |
| Kolleg/-innen berichten, dass sie von Klient/-innen nach NP gefragt werden.  Colleagues report that they are asked about INI by patients. |  |  |  |  |  |
| Ich selbst werde von Angehörigen explizit zu NP gefragt.  I myself am explicitly asked about INI by family-caregiver. |  |  |  |  |  |
| Kolleg/-innen berichten, dass sie von Klient/-innen nach NP gefragt werden.  Colleagues report that they are asked about INI by patients. |  |  |  |  |  |

## 2.3 Welche Meinung haben Sie zu folgenden Aussagen?

## 2.3 Regarding the following statements, what do you think??

## *Bitte in jeder Zeile eine Antwort auswählen.*

## *Please select one answer in each line.*

|  | stimme überhaupt nicht zu  Strongly disagree |  |  |  | stimme voll zu  Strongly agree |
| --- | --- | --- | --- | --- | --- |
| Eine Beratung zu NP gehört zu den Aufgaben der ambulanten Pflege.  Counselling about INI is one of the tasks of home nursing. |  |  |  |  |  |
| Anwendung von NP gehört zu den Aufgaben der ambulanten Pflege.  The use of INI is one of the tasks of home nursing. |  |  |  |  |  |
| Die Haltung meiner Kollegen bezüglich NP schätze ich eher positiv ein.  I think my colleagues‘ attitude towards INI is rather positive. |  |  |  |  |  |
| Die Haltung der Hausärzte bezüglich NP schätze ich eher positiv ein.  I think the attitude of family doctors towards INI is rather positive. |  |  |  |  |  |
| Pflegedienste in denen NP angewendet werden, sind besonders attraktiv für Pflegende.  Nursing services that use INI are particularly attractive to nurses. |  |  |  |  |  |

## 2.4 Welche Möglichkeit gibt es derzeit, naturheilkundliche Pflege finanziell abzurechnen?

## 2.4 What are the current options for reimbursing INI?

*Bitte geben Sie* ***alle*** *zutreffenden Antworten an.*

*Please indicate* ***all*** *appropriate answers.*

|  | Mir ist keine Möglichkeit bekannt  I am not aware of any possibility |
| --- | --- |
|  | Als zusätzliche privat zu zahlende Leistung  As an additionalm privately paid service |
|  | Als Leistung nach Gebührenordnung und zwar  As a service according to the fee schedule, namely |
|  | Sonstige und zwar  Other, namely |

## 3. Kenntnisse und Informationsbedarf

## 3. Knowledge and need for information

## 3.1 Wie gut fühlen Sie sich insgesamt zu naturheilkundlicher Pflege informiert?

## 3.1 How well informed do you feel about INI overall?

## *Bitte wählen Sie mit dem Schieberegler den entsprechenden Wert aus.*

## *Please use the slider to set the appropriate value.*

|  |  |  |
| --- | --- | --- |
|  |  |  |
| sehrschlecht |  | sehrgut |
| verybad |  | verygood |

## |______|______|______|______|______|______|______|______|______|

**_________________________________________________________________**

## 3.2 Welchen Stellenwert hatte naturheilkundliche Pflege in Ihrer Pflegeausbildung?

## 3.2 How relevant has INI been in your nursing education?

## *Bitte wählen Sie mit dem Schieberegler den entsprechenden Wert aus.*

## *Please use the slider to set the appropriate value.*

|  |  |  | |
| --- | --- | --- | --- |
|  |  |  | |
| überhaupt keinennone at all |  | einen sehr hohena very high | |
|  |  | |  |

## |______|______|______|______|______|______|______|______|______|

## 3.3 Zu welchen der genannten Methoden würden Sie gerne mehr wissen (z. B. im Rahmen von Fortbildungen)?

## 3.3 Which of the methods mentioned would you like to know more about (e.g. as part of further training)?

*Bitte wählen Sie* ***alle*** *zutreffenden Methoden aus.*

*Please select all applicable methods.*

| **Zu keinen**  **To none** |  |
| --- | --- |
| Akupressur  Acupressure |  |
| Anthroposophische Pflege  Anthroposophic nursing care |  |
| Aromapflege  Aromatherapy |  |
| *Wickel und Auflagen*  *Compresses* |  |
| *Kneipp`sche Anwendungen (Wechselbäder, Güsse)*  Kneipp-therapy |  |
| Rhythmische Einreibungen nach Wegmann/ Hauschka  Rhythmical embrocation according to Wegmann/Hauschka |  |
| *Inhalationen*  Inhalation |  |
| Reflexzonenmassage  Reflexology |  |
| Heilpflanzentee  Medicinal herbal tea |  |
| Andere: _________________________  Other: _________________________ |  |

**4. Demografische Angaben**

**4. Demographic data**

**4.1 Ihr Alter**

**4.1 Your age**

*Bitte geben Sie Ihr Alter in Jahren an, z.B. 43*

Please indicate your age in years, e.g. 43

_ _

**4.2 Ihr Geschlecht**:

**4.2 Your gender:**

|  | Männlich  male |
| --- | --- |
|  | Weiblich  female |
|  | Divers  diverse |

**4.3 Ihre berufliche Qualifikation**

**4.3 Your professional qualifications***

*Bitte geben Sie* ***alle*** *zutreffenden Antworten an.*

*Please select* ***all*** *appropriate answers.*

|  | Gesundheits- und Krankenpflege 3-jährig. Examiniert  Registered nurse (3-year qualification) |
| --- | --- |
|  | Gesundheits- und Krankenpflege primärqualifizierendes Studium  Bachelor of nursing |
|  | Kinder-Gesundheits- und Krankenpflege 3-jährig examiniert  Pediatric nurse (3-year qualification) |
|  | Kinder-Gesundheits- und Krankenpflege primärqualifizierendes Studium  Bachelor of nursing (pediatric) |
|  | Altenpflege 3-jährig examiniert  Geriatric nurse (3-year qualification) |
|  | Generalistische Pflegeausbildung 3-jährig examiniert  General nurse (3-year qualification) |
|  | Generalistische Pflegeausbildung primärqualifizierendes Studium  General nurse Bachelor of nursing |
|  | Pflegestudium und zwar  Nursing studies |
|  | Sonstige und zwar _________________________________________________  Other, namely _________________________________________________ |

^*^Nursing education in Germany is predominantly vocational, with few bachelor's degree programs. Some nurses also pursue a bachelor's or master's degree in nursing science.

**4.4. In welchem Jahr haben Sie das Pflegeexamen abgelegt?**

**4.4 What year did you take the nursing exam?**

_ _ _ _

**4.5 Haben Sie eine Zusatzqualifikation im Bereich NP?**

**4.5 Do you have an additional qualification in INI?**

*Bitte wählen Sie die zutreffende Antwort aus.*

*Please select the appropriate answer.*

|  | ja  yes |
| --- | --- |
|  | nein  no |

**4.5.1 Wenn ja, in welcher Methode?**

**4.5.1 If so, in which method?**

*Mehrfachauswahl möglich*

*Select all that apply*

| Akupressur  Acupressure |  |
| --- | --- |
| Anthroposophische Pflege  Anthroposophic nursing care |  |
| Aromapflege  Aromatherapy |  |
| *Wickel und Auflagen*  *Compresses* |  |
| *Kneipp`sche Anwendungen (Wechselbäder, Güsse)*  *Kneipp-therapy* |  |
| Rhythmische Einreibungen nach Wegmann/ Hauschka  Rhythmical embrocation according to Wegmann/Hauschka |  |
| *Inhalationen*  Inhalation |  |
| Reflexzonenmassage  Reflexology |  |
| Heilpflanzentee  Tea of medicinal plants |  |
| Andere: _________________________  Other: _________________________ |  |

**4.6 Welche Leitungsfunktion haben Sie?**

**4.6 What is your leadership role?**

*Bitte geben Sie* ***alle*** *zutreffenden Antworten an.*

*Please select* ***all*** *appropriate answers.*

|  | Pflegedienstleitung  Head of nursing service |
| --- | --- |
|  | Stellv. Pflegedienstleitung  Deputy head of nursing service |
|  | Geschäftsführung  Management |
|  | Sonstige und zwar__________________________________________  Other, namely __________________________________________ |
|  | Ich habe keine Leitungsfunktion.  I habe no leadership function. |
|  |  |

**4.7 Haben Sie Aufgaben mit direktem Patientenkontakt? (z.B. Pflegeanamnese, Pflegevisite, Beratung nach SGB IX § 37)**

**4.7 Do you have direct patient responsibilities? (e.g. assessment, education)**

|  | ja  yes |
| --- | --- |
|  | nein  no |

**4.8 Ihr Beschäftigungsumfang:**

**4.8 Your percentage of work:**

|  | unter 50%  under 50 % |
| --- | --- |
|  | 50-75% |
|  | 75-100% |

**5. Angaben zum ambulanten Pflegedienst**

**5. Details of the home nursing service**

**5.1 Standort der Ambulante Pflege:**

**5.1 Location of the home nursing service:**

**In welchem Gebiet ist der ambulante Pflegedienst hauptsächlich tätig?**

**What area does the home nursing service primarily serve?**

|  | Großstadt > 100.000 Einwohner  Big city > 100,000 inhabitants |
| --- | --- |
|  | Kleinstadt 15.000 bis 100.0000 Einwohner  Small town 15,000 to 100,000 inhabitants |
|  | Ländliches Gebiet < 15.0000 Einwohner  Rural area < 15,000 inhabitants |

**5.2 Wie viele Mitarbeitende hat der Pflegedienst?**

**5.2 How many employees does the home nursing service have?**

**Anzahl Personen gesamt**

**Total number of persons**

_ _ _

**5.3 Haben Sie Mitarbeitende mit Zusatzqualifikation in NP?**

**5.3 Do you have employees with additional qualifications in INI?**

|  | ja  yes |
| --- | --- |
|  | nein  no |

**5.3.1 Wenn ja, in welcher Methode?**

**5.3.1 If so, in which method?**

| Akupressur  Acupressure |  |
| --- | --- |
| Anthroposophische Pflege  Anthroposophic nursing care |  |
| Aromapflege  Aromatherapy |  |
| *Wickel und Auflagen*  *Compresses* |  |
| *Kneipp`sche Anwendungen (Wechselbäder, Güsse)*  *Kneipp-therapy* |  |
| Rhythmische Einreibungen nach Wegmann/ Hauschka  Rhythmical embrocation according to Wegmann/Hauschka |  |
| *Inhalationen*  *Inhalation* |  |
| Reflexzonenmassage  Reflexology |  |
| Heilpflanzentee  Tea of medicinal plants |  |
| Anderes: _________________________  Other: _________________________ |  |

**5.4 Welchen Träger hat Ihr Pflegedienst?**

**5.4 Which organization provides your home nursing service?**

*(Mehrfachauswahl ist möglich)*

*(multiple selection is possible)*

Arbeitgeber und Berufsverband Privater Pflege e.V. (ABVP)

Employers and Professional Association of Private Nursing (ABVP)

Baden-Württembergische Krankenhausgesellschaft (BWKG)

Baden-Württemberg Hospital Association (BWKG)

Bund privater Anbieter sozialer Dienste (bpa)

Association of private providers of social services (bpa)

Bundesverband Ambulante Dienste und Stationäre Einrichtungen e.V. (bad)

Federal Association of Outpatient Services and Inpatient Facilities e.V. (bad)

Bundesarbeitsgemeinschaft Hauskrankenpflege (BAH)

Federal Working Group for Home Nursing (BAH)

Caritasverband Erzdiözese Freiburg

Caritas Association Archdiocese of Freiburg

Caritasverband Erzdiözese Rottenburg-Stuttgart

Caritas Association Archdiocese of Rottenburg-Stuttgart

Diakonie Baden

Diaconia Baden

Diakonie Württemberg

Diaconia Württemberg

Verband der Deutschen Alten- und Behindertenhilfe e.V. (VDAB)

German Association for the Elderly and Disabled (VDAB)

Verband für anthroposophische Pflege (vfap)

Association for Anthroposophic Nursing (vfap)

Anderer und zwar

Other, namely

**6. Anmerkungen**

**6. Annotations**

### 6.1 Welche Herausforderungen gibt es aus Ihrer Erfahrung im Zusammenhang mit NP in der ambulanten Pflege?

**6.1** **What challenges have you encountered in your experience regarding INI in home nursing service?**

______________________________________________________________________________________________________________________________________________________________________________________________________________________________

**6.2 Haben Sie noch Anmerkungen zur Studie?**

**6.2 Do you have any comments about this study?**

____________________________________________________________________________________________________________________________________________________

__________________________________________________________________________

## Wir freuen uns über Ihre Rückmeldung!

## Your feedback is appreciated!

E**ndseite**

**Final page**

**Vielen Dank für Ihre Teilnahme an der Studie!**
Ihre Angaben wurden gespeichert. Sie können das Fenster jetzt schließen.

**Thank you for participating in this study!**

Your information has been saved. You can now close this window.

**Sie haben noch Fragen oder möchten über die Ergebnisse der Befragung informiert werden?**Sie können sich gerne an uns wenden.

**Do you have any questions or would you like to be informed about the results of the survey?**

Feel free to contact us.

- Regina Stolz M.A. Pflegewissenschaft, Doktorandin ([regina.stolz@med.uni-tuebingen.de](mailto:regina.stolz@med.uni-tuebingen.de))
- Prof. Dr. med. Stefanie Joos, Ärztliche Direktorin, Institut für Allgemeinmedizin und Interprofessionelle Versorgung, Tübingen ([stefanie.joos@med.uni-tuebingen.de](mailto:stefanie.joos@med.uni-tuebingen.de))

Regina Stolz M.A. Nursing Science, PhD student (regina.stolz@med.uni-tuebingen.de)

Prof. Dr Stefanie Joos, Medical Director, Institute for General Practice and Interprofessional Care, Tübingen (stefanie.joos@med.uni-tuebingen.de)
